# Stents Eluting 6-Mercaptopurine Reduce Neointima Formation and Inflammation while Enhancing Strut Coverage in Rabbits

**DOI:** 10.1371/journal.pone.0138459

**Published:** 2015-09-21

**Authors:** Matthijs S. Ruiter, Claudia M. van Tiel, Albert Doornbos, Goran Marinković, Aart C. Strang, Nico J. M. Attevelt, Vivian de Waard, Robbert J. de Winter, Rob Steendam, Carlie J. M. de Vries

**Affiliations:** 1 Department of Medical Biochemistry, Academic Medical Center, University of Amsterdam, Amsterdam, The Netherlands; 2 InnoCore Pharmaceuticals, Groningen, The Netherlands; 3 Department of Vascular Medicine, Academic Medical Center, University of Amsterdam, Amsterdam, The Netherlands; 4 Central Laboratory Animal Research Facility, Faculty of Veterinary Medicine, University of Utrecht, Utrecht, The Netherlands; 5 Department of Cardiology, Academic Medical Center, University of Amsterdam, Amsterdam, The Netherlands; North Carolina A&T State University, UNITED STATES

## Abstract

**Background:**

The introduction of drug-eluting stents (DES) has dramatically reduced restenosis rates compared with bare metal stents, but in-stent thrombosis remains a safety concern, necessitating prolonged dual anti-platelet therapy. The drug 6-Mercaptopurine (6-MP) has been shown to have beneficial effects in a cell-specific fashion on smooth muscle cells (SMC), endothelial cells and macrophages. We generated and analyzed a novel bioresorbable polymer coated DES, releasing 6-MP into the vessel wall, to reduce restenosis by inhibiting SMC proliferation and decreasing inflammation, without negatively affecting endothelialization of the stent surface.

**Methods:**

Stents spray-coated with a bioresorbable polymer containing 0, 30 or 300 μg 6-MP were implanted in the iliac arteries of 17 male New Zealand White rabbits. Animals were euthanized for stent harvest 1 week after implantation for evaluation of cellular stent coverage and after 4 weeks for morphometric analyses of the lesions.

**Results:**

Four weeks after implantation, the high dose of 6-MP attenuated restenosis with 16% compared to controls. Reduced neointima formation could at least partly be explained by an almost 2-fold induction of the cell cycle inhibiting kinase p27^Kip1^. Additionally, inflammation score, the quantification of RAM11-positive cells in the vessel wall, was significantly reduced in the high dose group with 23% compared to the control group. Evaluation with scanning electron microscopy showed 6-MP did not inhibit strut coverage 1 week after implantation.

**Conclusion:**

We demonstrate that novel stents coated with a bioresorbable polymer coating eluting 6-MP inhibit restenosis and attenuate inflammation, while stimulating endothelial coverage. The 6-MP-eluting stents demonstrate that inhibition of restenosis without leaving uncovered metal is feasible, bringing stents without risk of late thrombosis one step closer to the patient.

## Introduction

Atherosclerotic disease of the coronary arteries can lead to reduced perfusion and even myocardial infarction. Percutaneous coronary intervention (PCI) has become an important alternative to invasive surgery, and is now one of the most common medical interventions [1]. In the Netherlands, 92% of all therapeutic PCI are stent implantations [2]. The introduction of drug-eluting stents (DES) has dramatically reduced restenosis rates compared to bare metal stents (BMS). In 2010 in the United States, 75% of the stents implanted during PCI were DES, against 25% BMS [3]. In current DES, late and very late stent thrombosis of 0.6–0.7% remain a safety concern, necessitating prolonged dual anti-platelet therapy [4]. Uncovered stent struts remain the primary substrate for stent thrombosis following DES implantation [5,6]. Delayed endothelialization can be induced by inflammatory response to the durable polymer coatings or by the used drug [5,7]. The early reports prompted attempts to replace the durable polymer coatings of first generation DES by bioresorbable polymer coatings [8], that would degrade completely after release of anti-restenotic drugs, leaving a bare metal stent with a proven long-term biocompatibility and safety profile [9]. Biodegradable urethane-linked polyetherester multi-block copolymers have been reported to exhibit the chemical and mechanical properties and vascular biocompatibility that are required for application as biodegradable DES coatings [10].

Currently applied drugs such as paclitaxel, rapamycin, zotarolimus and everolimus are anti-proliferative regardless of cell type, thereby effectively reducing smooth muscle cell (SMC) proliferation, yet negatively affecting endothelialization of stent struts. Cell-specific therapy may prevent this complication, giving rise to safer stents. Nuclear receptor Nur77, an orphan nuclear receptor of the NR4A subfamily, also referred to as NR4A1, TR3, NGFI-B or NAK-1, is involved in cellular processes such as proliferation, migration, apoptosis and differentiation [11–14]. Nur77 has been shown to have beneficial effects on the vessel wall in a cell-specific fashion. Firstly, Nur77 was shown to prevent SMC proliferation in vitro, and to induce a more quiescent SMC phenotype in vivo [15–17]. Secondly, activation of Nur77 promotes survival of endothelial cells and capillary sprouting [18,19]. Thirdly, Nur77 is involved in bone marrow differentiation and reduces the inflammatory response [20–22]. Together, these functions protect against neointima formation and atherosclerosis in vivo [17,20], and provide an interesting approach for prevention of stent restenosis and thrombosis. 6-Mercaptopurine (6-MP) is the active metabolite of the immunosuppressive drug azathioprine and is a well-documented activator of Nur77, with demonstrated in vitro and in vivo beneficial effects on vascular cells [18,23] and may be the key to safer DES. 6-MP has been administered in different animal models, with different techniques and concentrations, and for different purposes. Systemic administration in animals is typically in the mg kg^-1^ day^-1^ range, similar as the immunosuppressive dose administrated in human [24]. In cell culture, concentrations are in the μM range, typically 10–50 μM [17,25]. The amount of 6-MP loaded on stents was furthermore based on our experience with drug-eluting ‘cuffs’ in mice, which released 10 or 30 μg over the course of 4 weeks [26]. Altogether, the 30 μg dose was chosen as the low dose and the high dose of 300 μg was determined by the highest loading at which controlled release could be established without making the coating too bulky or prone to deformation upon crimping or expanding.

In the present study, we examine the suitability of biodegradable urethane-linked multi-block copolymers for development of 6-MP eluting coronary stents and show that these novel 6-MP eluting biodegradable polymer coated coronary stents reduce restenosis by inhibiting SMC proliferation and reducing inflammation, without negatively affecting endothelialization of the stent surface.

## Materials and Methods

### Stent Coating

Using an ultrasonic spray-coating process (MedicoatMicromist system, Sono-Tek, Milton USA), Kaon 3.0x15 mm balloon expandable cobalt chromium stents (Fortimedix, Nuth, the Netherlands) were spray-coated with blends of SynBiosys GLL, a multiblock copolymer consisting of 50% w/w of poly(DL-lactide-co-glycolide) and 50% w/w of poly(DL-lactide) and SynBiosys GPCGL, a multiblock copolymer consisting of 15% w/w of poly(glycolide-PEG600-ε-caprolactone) and 85% w/w of poly(DL-lactide-co-glycolide) (InnoCore Pharmaceuticals, Groningen, the Netherlands) containing 0, 7.5 or 33wt.% 6-MP (purity >99.5%, Acros Organics). Coating formulations are presented in Table 1. Coating solutions were prepared by dissolving the required amounts of polymers and 6-MP in anhydrous tetrahydrofuran (Sigma) at a polymer concentration of approximately 1%. Prior to spray-coating all solutions were filter sterilized using an 0.2 μm PTFE filter (Sartorius). Excess solvent was removed from the coatings by first drying in a purged nitrogen environment followed by drying under vacuum. 6-MP loads were targeted at 30 and 300 μg per stent, respectively. Coated Stents were mounted on a stent delivery system (Clearstream DAC 135 3.0x15mm balloon catheter), crimped in place at room temperature with an automated crimping tool (Fortimedix, Nuth, The Netherlands) and E-beam sterilized at BGS Beta Gamma Service GmbH & Co.KG (Sits Wiehl, Germany) at 10MeV with a minimal dose of 25 kGy.

**Table 1:** Coating formulations.

*6-MP (*μ*g)*	*Coating (*μ*g)*	*Coating Composition*	*Polymer (*μ*g)*	*6-MP (%)*
0	400	GLL/GPCGL 50/50%	400	0
30	400	GLL/GPCGL 75/25%	370	7.5
300	900	GLL/GPCGL 50/50%	600	33

6-MP, 6-mercaptopurine; GLL, multiblock copolymer 50% (w/w) poly(DL-lactide-co-glycolide), 50% (w/w) poly(DL-lactide); GPCGL, multiblock copolymer 15% (w/w) poly(glycolide-PEG600-ε-caprolactone), 85% (w/w) poly(DL-lactide-co-glycolide)

### Coating quality

Coated stents were examined with optical and scanning electron microscopy (SEM) to assess the coating quality and check for potential damage to the coating following the crimping procedure. To gently retrieve coated stents from their delivery systems, crimped stents were expanded by immersing the stent/balloon assembly for 1 min in phosphate/citric acid buffered release medium (200 mM, pH 7.4) at 37°C, followed by inflation of the balloon at a pressure of 9 atm whereafter the stent could be removed from the balloon. Scanning Electron Microscopy (SEM) imaging of pre-expanded and expanded stents of each group was performed with a Jeol JSM 6301F to study coating integrity and homogeneity. Prior to SEM evaluation, stents were sputter-coated with a Pd-Au layer.

### 6-MP elution in vitro

Release of 6-MP from coated stents was studied in triplicate under sink conditions in 5 mL phosphate/citric acid release buffer (200mM, pH 7.4, 0.02% sodium azide) incubated at 37°C under mild shaking using a shaking water bath. At the start the stent/balloon catheter assembly was immersed in the release medium for 1 minute whereafter stents were expanded by inflation of the balloon. After various time intervals, the concentration of 6-MP in the release medium was quantified by HPLC, whereafter the entire volume of buffer was refreshed. HPLC was performed on a Waters 2695 Alliance system (Etten-Leur, The Netherlands) consisting of a 2998 Photodiode array detector and a computer with Empower 2 Software. Prior to analysis, the samples were filtered over an 0.2μm PTFE filter. Separation was performed using a Symmetry C18 (4.6×150mm, 5μm) reverse phase column (Waters, Etten-Leur, The Netherlands) at 30°C using a flow rate of 1.0mL/min and an injection volume of 20μL. The mobile phase was 10mM phosphate buffer pH 2.25:acetonitrile:methanol (96:3:1 v/v). 6-MP was detected by UV absorption at a wavelength of 324nm. The standard curve of 6-MP was established and the concentration of unknown samples was calculated from the standard curve. The linearity was 0.9998 in the range of 0.05μg/g to 50μg/g. The retention time of 6-MP was 3.8 minutes.

### 6-MP activity in vitro

To establish 6-MP stability over time, 6-MP activity from coated stents was measured 4 and 16 months after production. 6-MP was dissolved from the stents in DMSO, diluted in H_2_O to a concentration of 200μM 6-MP and subsequently diluted in medium to obtain a 6-MP concentration of 50 μM. The medium was added to C2C12 cells transfected with Nur77, the NurRE luciferase reporter JA983 [27] and a plasmid encoding renilla luciferase as a control for transfection efficiency. After 24 hours of 6-MP treatment, the cells were lysed and luciferase and renilla activity in the cells was measured in triplicate by luminescence with the dual-luciferase reporter assay (Promega) according to the manufacturer’s protocol on a luminometer (GloMax, Promega).

### Animal model

Animal care and experimental procedures were reviewed and approved by the Institutional Animal Care and Research Committee at the Academic Medical Center, Amsterdam, The Netherlands (protocol number DBC 102110) and conform to the Directive 2010/63/EU of the European Parliament on the protection of animals used for scientific purposes. In this study, 17 male New Zealand White rabbits, weighing 2.7–3.1 kg were housed individually, were fed rabbit chow and were given access to drinking water ad libitum. After surgery, animals were treated with aspirin (38 mg/day) dissolved in drinking water.

### Surgical Procedure

Animals were anesthetized with ketamine (15mg/kg) and dexmedetomidine (0.20mg/kg) injection. Prophylactic antibiotics (Baytril, 5 mg/kg intravenously) were administered before and 1 day after the operation. After intravenous heparin administration (100IU/kg), the left carotid artery was approached surgically and accessed with a 5F introducer sheath (Cordis, Miami Lakes, Fl, USA). Roadmap angiography was performed by injecting contrast agent (Hexabrix, 320mg I/ml) diluted 1:1 with saline. Balloon-induced endothelial cell denudation of the iliac artery was subsequently performed towards the aorta on both sides with a balloon catheter (Abbott, Abbott Park, Il, USA) inflated to 11 atm. Stents were deployed (single inflation of 9 atm for 10 sec) with an oversize ratio of 1.1 to 1.2. Stents of the different groups were randomly assigned to animals and arteries. After stent placement, the carotid artery was sutured and the wound was closed, and animals received buprenorphine (Temgesic, 0.03mg/kg). For quantitative morphometric analysis stented arteries were harvested after 4 weeks. For short-term qualitative analysis, 300 μg 6-MP eluting stents and conventional Cypher® rapamycin-eluting stents (Cordis, Miami Lakes, Fl, USA) were implanted, and harvested after 1 week. At the end of the experiment, animals were anesthetized as before. By medial laparotomy the abdominal aorta was accessed to perform control angiography. Subsequently, the animal was euthanized with a lethal dose of pentobarbital.

### Sample processing

Stented arterial segments were harvested after 4 weeks for quantitative analysis (3 groups, n = 10 per group), or after 1 week (2 groups, n = 2 per group) for qualitative analysis with SEM. Segments harvested 4 weeks after implantation were fixed under pressure with 4% formaldehyde, fixed overnight in 4% formaldehyde after excision, and stored in 70% ethanol. The segments were dehydrated in a graded series of acetone and embedded in resin (methyl methacrylate and butyl methacrylate, 1:1). Sections (7μm) were cut with a rotary microtome (Leica) from the middle of the stent, after sawing the segment with a band saw (Exakt). Segments harvested after 1 week were fixed in a mixture of 1% (wt/vol) glutaraldehyde and 4% (wt/vol) formaldehyde in 100mmol/L sodium cacodylate buffer (pH 7.4) and incubated at 4°C. Stent were cut longitudinally for *en face* imaging. After dehydration in a graded series of ethanol followed by hexamethyldisilazane, segments were sputtered with a gold layer.

### Histomorphometric analysis

Sections were attached to glass slides and dried O/N. General histology was determined with hematoxylin and eosin (HE) staining. Morphometric analysis and injury score were performed on sections stained with Lawson-Van Gieson staining. Injury score was performed according to Schwartz, wherein the injury is scored with 0 (no injury); 1 (internal elastic lamia lacerated); 2 (media injury) or 3 (laceration of external elastic lamia) [28]. The lumen cross-sectional area, external elastic lamina (EEL) area and internal elastic lamina (IEL) area corrected for strut holes were assessed with imaging software (Leica Qwin). The percentage of stenosis was calculated as [1-(lumen area/IEL area)]*100. Additionally, Masson’s Trichrome staining was performed to visualize the medial SMC layer and the adventitial collagen layer.

### Immunohistochemistry

Sections were stained immunohistochemically using antibodies against smooth muscle α-actin (1A4, DAKO), macrophages (RAM11, DAKO), cleaved caspase-3 (Asp175, Cell Signaling), PCNA (BD) and p27^Kip1^ (Abcam), followed by horseradish peroxidase (HRP)-conjugated goat anti-mouse antibodies (Southern Biotech) or poly HRP-anti-rabbit IgG (Immunologic, Duiven, the Netherlands) followed by 3,3-diaminobenzidine (DAB) substrate color development (Immunologic). Inflammation score according to Kornowski was determined on RAM11-stained sections, allocating values of 0 (none), 1 (mild with minimal infiltrated inflammatory cells), 2 (moderate) or 3 (severe, with large clusters of inflammatory cells with granulomatous morphology) [29]. Cleaved caspase-3, PCNA and p27^Kip1^ quantification was performed on 4 areas per stent section for all stents and expressed as positive area of the neointima. Additionally, the number of adventitial capillaries was counted.

### Electron microscopy

Segments for SEM analysis were visualized at the Van Leeuwenhoek Center for Advanced Microscopy, Department of Cell Biology, Academic Medical Center Amsterdam, The Netherlands.

### Statistical analysis

Numerical data are presented as mean±standard error. Differences in 6-MP stability were compared with ANOVA. Comparisons in morphometry between the 3 drug loading groups were tested with Jonkheere’s independent samples trend test. Mann-Whitney U-test was used for comparisons of either DES versus control, and to compare groups in IHC analysis. P<0.05 was considered statistically significant.

## Results

### Stent coating released 6-MP from a smooth surface

**Fig 1:**
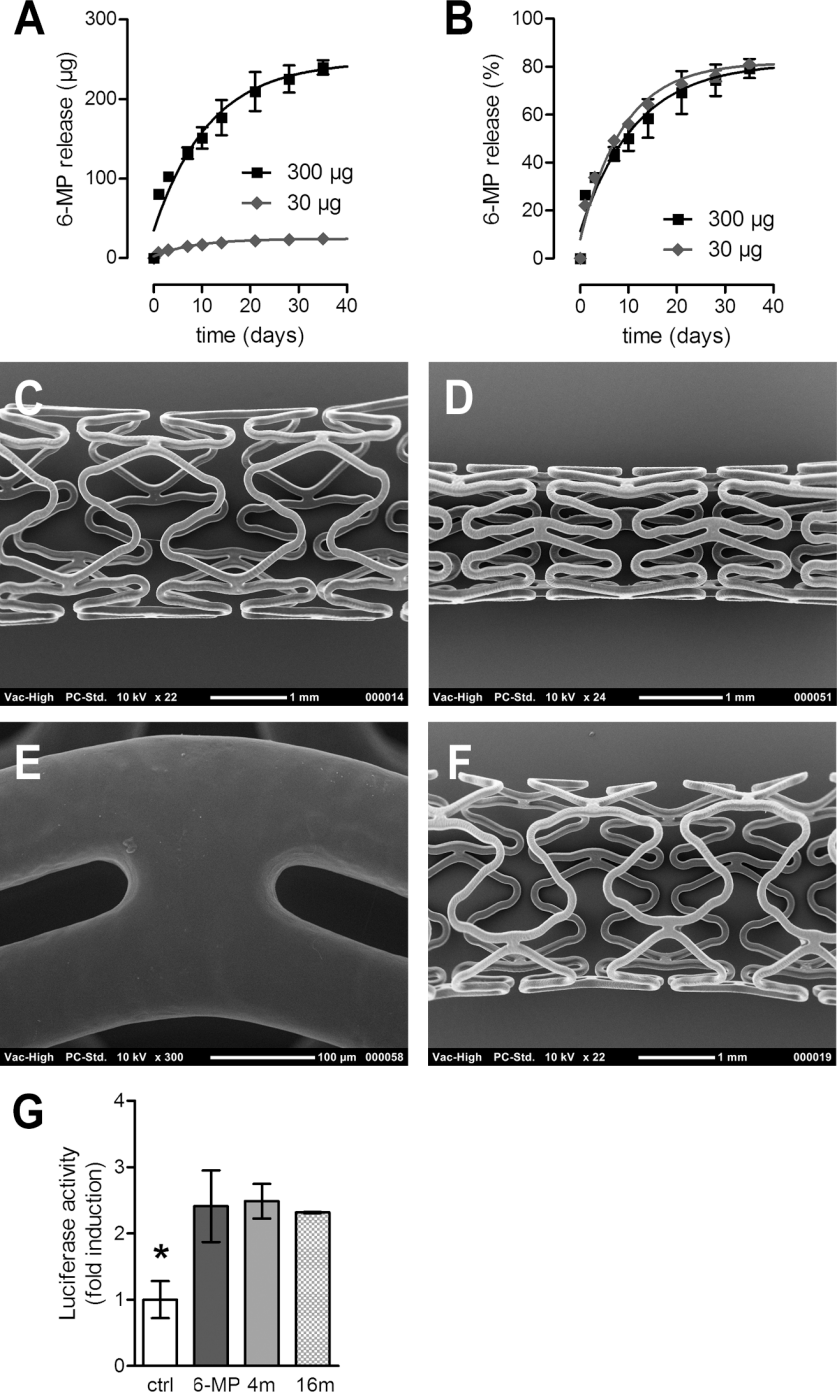
Characteristics of 6-MP eluting stents: 6-MP release, coating quality, and biological activity of eluted 6-MP. The cumulative release of 6-MP was assayed in vitro for the 30 μg DES (diamonds, n = 3) and the 300 μg 6-MP eluting stents (squares, n = 3) up to 38 days, and expressed as absolute release (A) as well as percentage release (B). A polymer-only stent (C) and a stent loaded with 300 μg 6-MP before (D,E) and after expansion (F) show smooth coating surfaces by SEM. The stability of 6-MP after storage was measured after elution from the stents and quantified by its ability to activate Nur77 in a luciferase reporter assay (G). 6-MP was retrieved from stents after 4 (4m, n = 6 measurements) and 16 months (16m, n = 6) and the activity was compared with a negative control (white bar, n = 9) and freshly dissolved 6-MP at 50 μM (black bar, n = 9). * depicts value that is statistically different from other groups analyzed by ANOVA. Error bars represent standard error.

Stent coating released 6-MP from a smooth surfaceTo assess the 6-MP release kinetics of the SynBiosys®-GLL / SynBiosys® GPCGL multi-block copolymer-based spray-coated stents with distinct doses of 6-MP, *in vitro* release profiles were analyzed. Stent coatings released 6-MP gradually according to first order release kinetics, delivering over 75% of the drug within one month (Fig 1A). The cumulative release profile (%) was similar for both 6-MP doses (Fig 1B). The quality of the coating was analyzed by scanning electron microscopy and the coating was shown to be evenly distributed over the stent, resulting in a smooth surface without webbing. In order to assess the mechanical performance of the coating, coated stents were mounted onto a balloon catheter, soaked in PBS at 37°C, and expanded by balloon inflation. Fig 1 shows a polymer-only stent (C) and a stent loaded with 300μg 6-MP before (D, E) and after expansion (F). The crimping and expansion process did not generate any damage to coatings. Coatings of expanded polymer-only and 6-MP eluting stents (30 and 300 μg) were smooth without any signs of cracking or delamination (Fig 1C and 1F).

To assess whether 6-MP eluted from the stents is biologically active after short and long-term storage, the biological activity of 6-MP eluted from the stents after storage for 4 months and 16 months (at 4°C) was tested. 6-MP enhanced the transcriptional activity of the nuclear receptor Nur77, which can be monitored with luciferase reporter assays. C2C12 cells were transfected with Nur77 and a luciferase reporter plasmid and subsequently the cells were stimulated and luciferase activity was measured. 6-MP eluted from the stents was just as potent as freshly dissolved 6-MP in enhancing Nur77 activity (Fig 1G). Based on these data we conclude that the 6-MP eluting stents exhibit the required release profiles from a smooth coating and that the compound is stable after prolonged storage.

### 6-MP inhibits neointima formation

**Fig 2:**
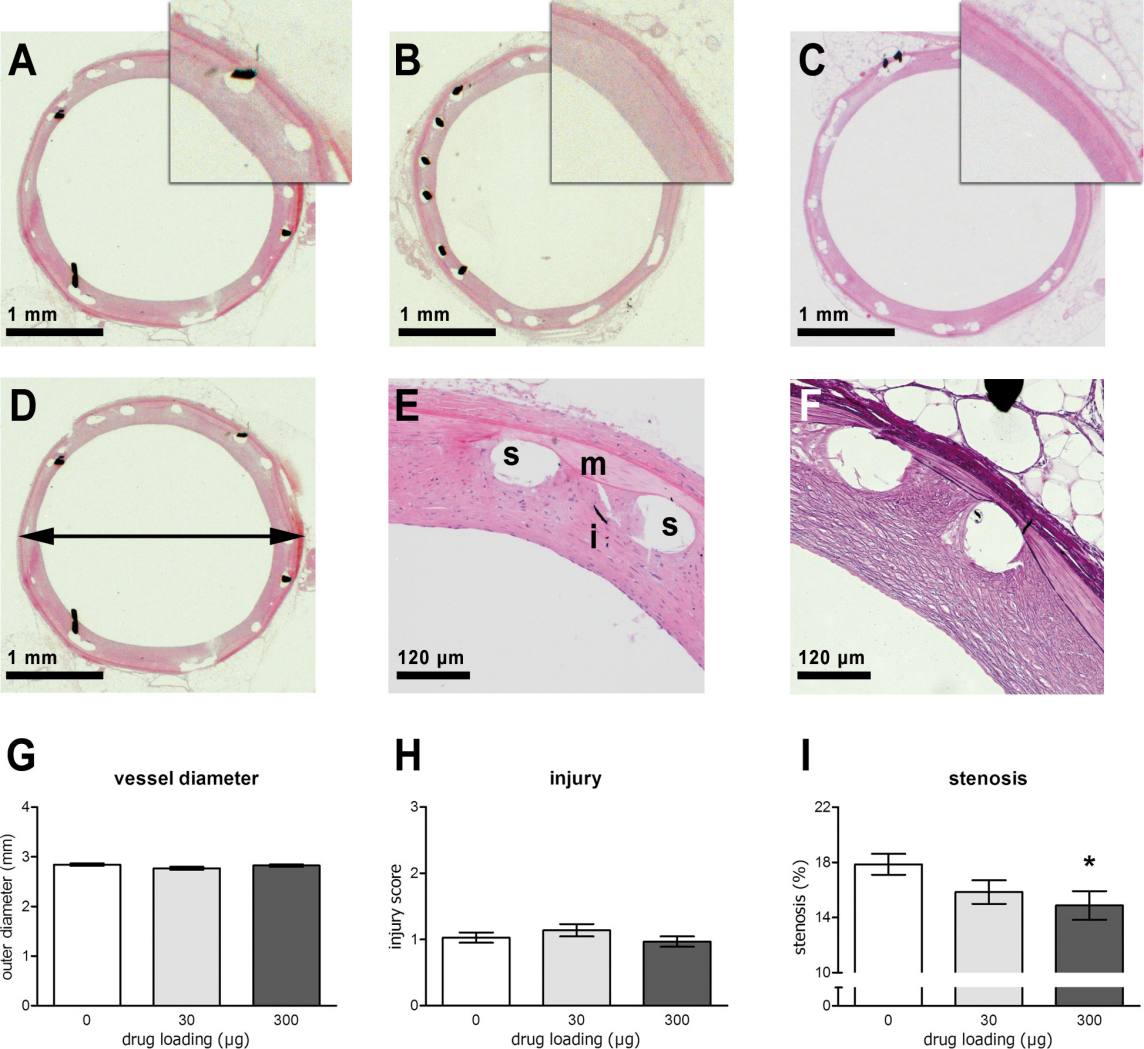
Morphology and morphometric analyses of sectioned arteries stented for 4 weeks. Representative HE-stained sections of arteries with stents eluting 0 (A), 30 (B) or 300 μg 6-MP (C). HE staining at low (D) and high (E) magnification with adventitia (a), media (m) and intima (i); strut holes are indicated by (s). Lawson-Van Gieson staining clearly reveals the lamina and thus the different layers of the vessel wall (F). The outer diameter of the stented arteries (G) was measured and found to be highly similar within and between the groups, indicating high reproducibility of stent implantation and expansion. The injury score was low in all stents and similar between groups (H). Lumen stenosis (I) was significantly different across the groups (Jonkheere’s independent samples trend test), with 16% reduction of stenosis in the 300 μg 6-MP eluting stents compared with the polymer-only group (Mann-Whitney U test). N = 10 for 0 and 30 μg, N = 9 for 300 μg. * depicts value that is statistically different from other groups. Error bars represent standard error.

To assess the effect of 6-MP eluting stents on vascular lesion formation, the stents. The stents implanted in iliac arteries of rabbits after local balloon injury were patent directly after deployment and just before harvest, as observed by angiography. One stent was damaged during postmortem processing and was excluded from further analysis. After sectioning of the plastic-embedded stented artery fragments, general histology was performed on tissue sections (representative pictures are shown in Fig 2A–2C). HE-stained sections reveal nuclei and cytoplasmic staining and showed intact adventitia and media, which was not damaged by the implanted stents (Fig 2D and 2E). Generally, neointima formation was evenly distributed. Lawson-van Gieson staining showed the elastic laminae and connective tissue and was performed to distinguish the different vascular layers more accurately for quantification purposes (Fig 2F).

Morphometric analyses were performed to determine the outer diameter of the stented vessels, as well as the extent of in-stent stenosis. Reproducibility of stent expansion was confirmed by assessment of the vessel outer diameter, demonstrating no differences between the groups (Fig 2G). The injury score, a semi-quantitative measure for the amount of injury to the vessel wall, was minimal and similar in all groups (Fig 2H). The main outcome of this study is the extent of lesion formation, which was significantly decreased with increasing 6-MP loading of the stents (Fig 2I). Stenosis, as calculated by percent of luminal occupancy, was significantly lower in the 300 μg 6-MP group (14.9±1.0) as compared to the control group (17.9±0.8; P = 0.02), whereas stenosis in the 30 μg 6-MP group (15.9±0.9) was not statistically different from the control.

### 6-MP does not affect apoptosis and enhances expression of the cell cycle inhibitor p27^Kip1^

**Fig 3:**
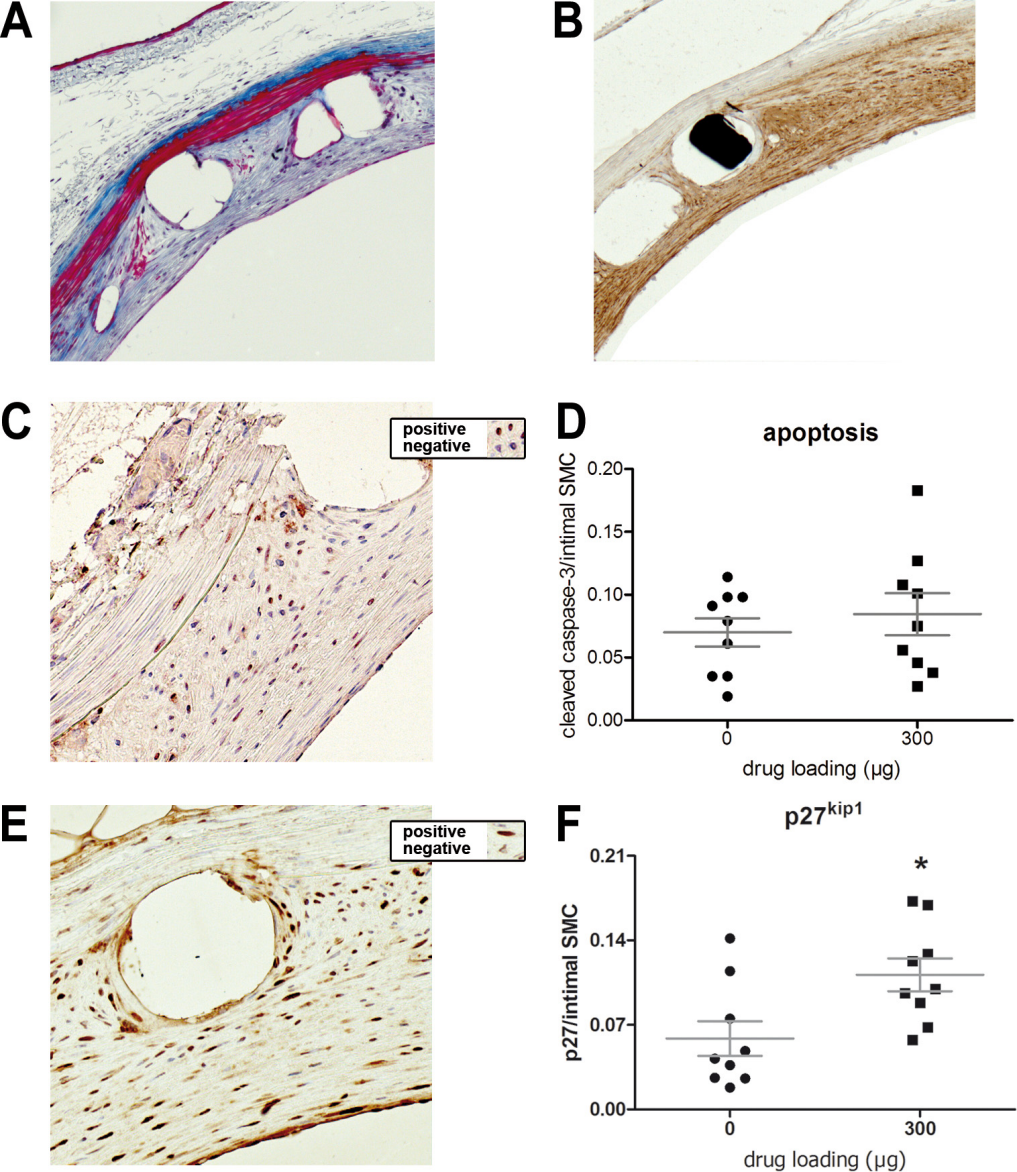
SMCs, apoptosis and expression of the cell cycle inhibitor p27^Kip1^ in arteries stented for 4 weeks. Masson trichrome staining (A) revealed collagen (blue), SMCs (red) and nuclei (black). The lesion predominantly consists of SMC, as was visualized by immunohistochemistry with the 1A4 antibody directed against SM-α actin (B). To assess the extent of apoptosis in the stented vessel segments, immunohistochemistry for cleaved caspase-3, an apoptosis marker, was performed (C, magnification 200x). Examples of positive and negative cells are presented in the insert. Apoptosis was low and similar between the groups (D). As a marker of cell cycle inhibition, p27^Kip1^ positive cells were shown by immunohistochemistry (E, magnification 200x, examples shown in insert). The ratio of positive cells was significantly higher in the high dose group compared to controls (F). PCNA was used as a marker for proliferation (G). No differences were found between the groups (H). N = 10 for 0 μg, N = 9 for 300 μg. Lines and error bars depict mean and standard error, respectively. * indicates significant difference with control group (Mann-Whitney U test).

In Masson’s Trichrome staining the medial SMC layer is intense red, and can be clearly distinguished from the neointima (Fig 3A). In addition, the SM-α actin staining showed that both the media and neointima consist predominantly of SMCs (Fig 3B). To elucidate the mechanism by which 6-MP inhibits neointima formation, immunohistochemistry was performed on sections of the high 6-MP dose compared with polymer-only sections. Reduced lesion formation may be explained by enhanced apoptosis of SMCs, therefore the expression of cleaved caspase-3 was quantified. No differences were observed between the groups, thus we concluded that 6-MP does not affect apoptosis in the vessel wall (Fig 3C and 3D). Alternatively, the growth of SMCs may be reduced by 6-MP, which has been shown to increase the expression of cell cycle inhibitors such as p27^Kip1^ in cultured SMCs. In line with the *in vitro* data, we observed significantly higher p27^Kip1^ expression (P = 0.02) in SMCs of the 6-MP group (112*10^−3^±14*10^−3^) compared to the polymer only group (58.7*10^−3^±14.4*10^−3^; Fig 3E and 3F). Additionally, expression of the proliferation marker PCNA was measured. No difference was observed between the groups, which may be explained by the time point of harvesting. Thus, without differences in expansion or injury of the vessel, 6-MP reduced stenosis, at least partly via enhanced expression of the cell cycle inhibiting kinase p27^Kip1^.

### 6-MP reduces tissue inflammation

**Fig 4:**
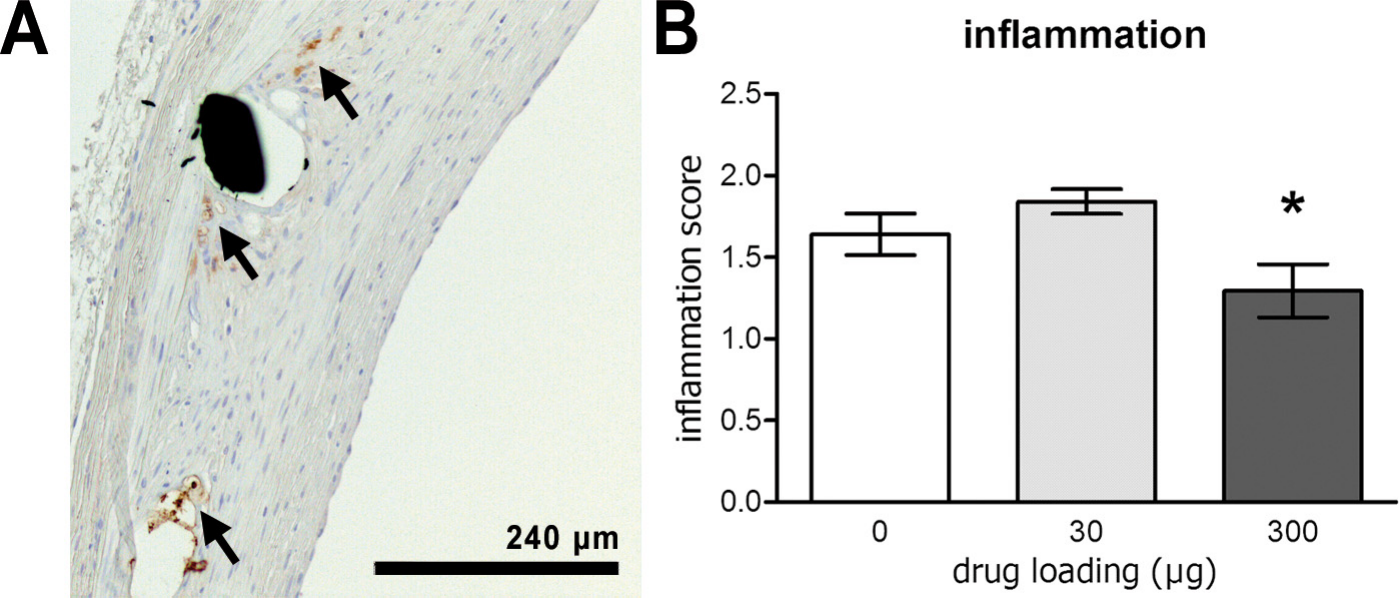
6-MP inhibits inflammation in the lesion. Macrophages, visualized in sections by immunohistochemistry with the RAM-11 antibody, were mainly localized around stent struts (A), as indicated by the arrows. The inflammation score was significantly lower in the high dose stents compared with the controls (B). White bars depict polymer-only (n = 10), light bars 30 μg 6-MP eluting stents (n = 10), dark bars 300 μg 6-MP eluting stents (n = 9). Error bars depict standard error, * indicates significant difference compared to control group (Mann-Whitney U test).

Inflammation in the vessel wall potentiates neointima formation and involves infiltration of macrophages. To quantify inflammation, sections were stained for macrophages (Fig 4A). Macrophages were observed in the tissue near the stent struts. In most sections single cells or small groups of macrophages were observed, whereas large aggregations of inflammatory cells were rare. The inflammation score was significantly decreased with increasing 6-MP loading (P = 0.03; Fig 4B). In conclusion, 6-MP, which is known for its immunosuppressive function, inhibits infiltration of macrophages into the vessel wall possibly leading to reduced activation of SMCs.

### 6-MP is beneficial for strut coverage

**Fig 5:**
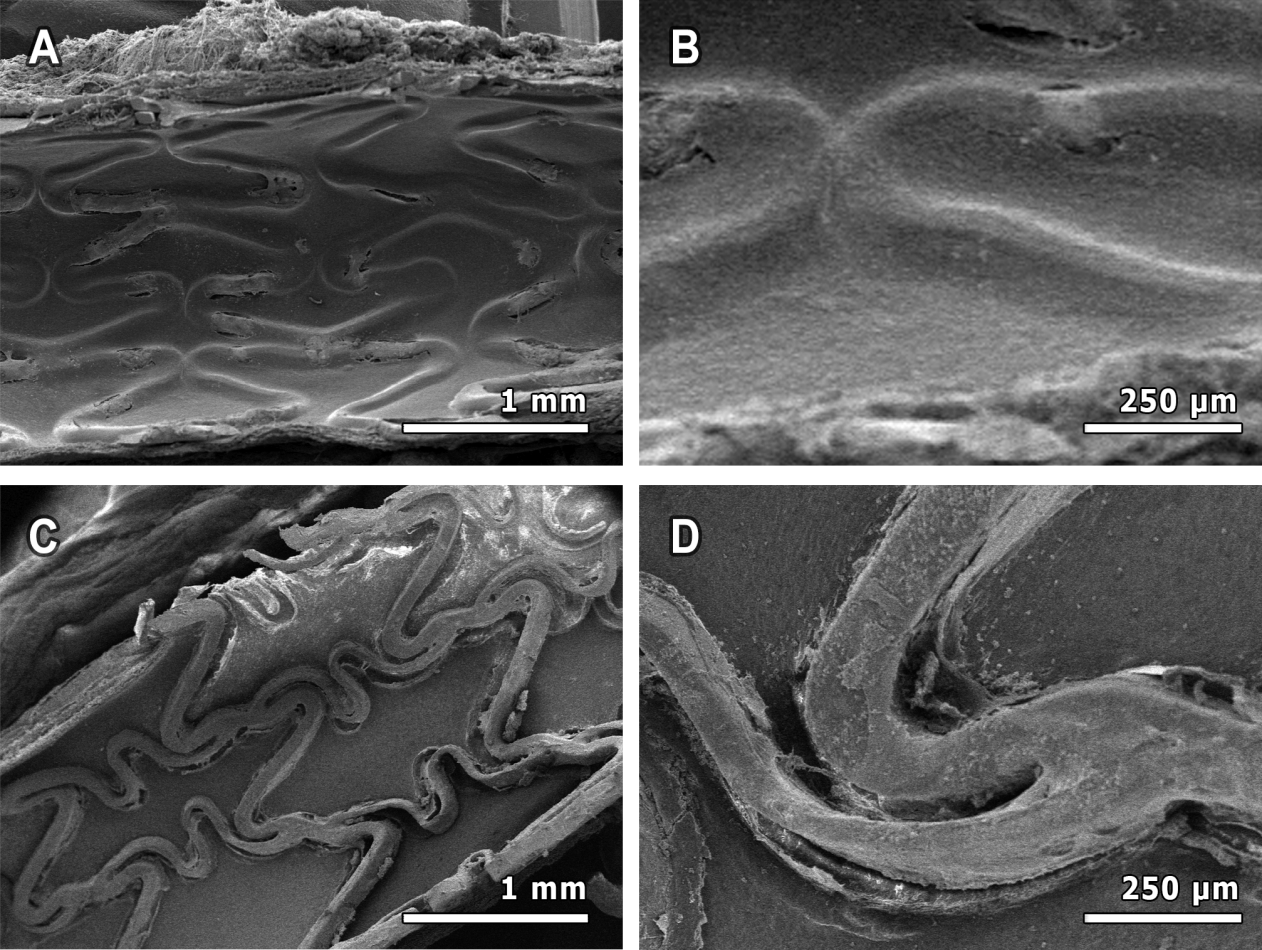
Scanning electron microscopy of stent surface 1 week after implantation. For qualitative *en face* assessment of stent coverage, high dose 6-MP eluting stents as well as Cypher rapamycin-eluting stents were implanted (N = 2). Stents excised after one week were cut longitudinally and sputtered with gold particles. 6-MP eluting stents (A,B) show good coverage, as opposed to rapamycin-eluting stents (C, D), which are completely exposed.

Endothelialization of stents reduces thrombosis risk and inhibits SMC proliferation. Given that 6-MP promotes endothelial cell survival, the coverage of stent surfaces was evaluated qualitatively by scanning electron microscopy. The surface of the 6-MP eluting stents was evenly covered by an endothelial cell monolayer and hardly any metal was exposed one week after implantation (Fig 5A and 5B), whereas the metal surface of the rapamycin-coated stent was completely exposed. (Fig 5C and 5D). This analysis revealed the crucial, beneficial difference between 6-MP eluting stents and rapamycin-eluting stents on endothelial cell recovery. 6-MP is thus beneficial for coverage of stents struts.

### 6-MP increases number of capillaries around rabbit iliac arteries

**Fig 6:**
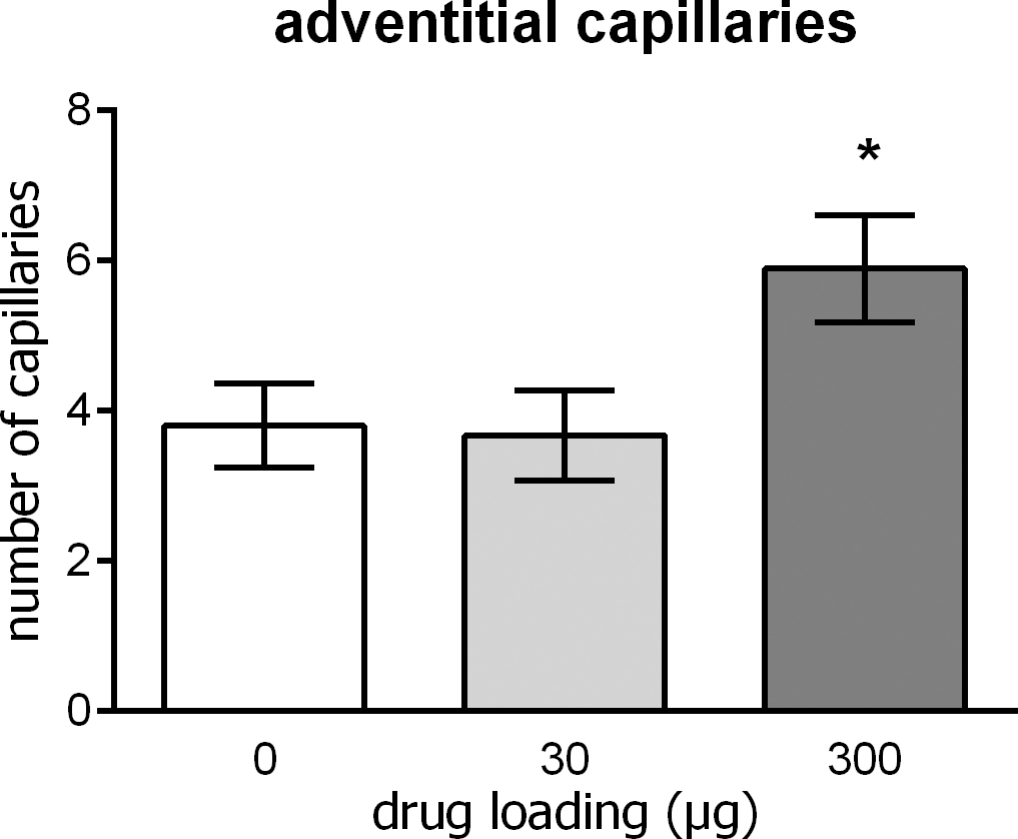
Adventitial capillaries. Higher 6-MP release resulted in higher numbers of capillaries (Jonkheere’s independent samples trend test, P = 0.02). Stents loaded with 300 μg 6-MP (n = 9) induced 30% more capillaries than polymer-only stents (N = 10, Mann-Whitney U test, P = 0.02). Low 6-MP dose (N = 10) failed to increase the number.

The number of capillaries was counted in sections of the stented vessels to determine the effect of 6-MP on angiogenesis. Hardly any capillaries were found in the thin, dense layer of adventitia, so capillaries adjacent to the adventitia were counted. Overall, high 6-MP release resulted in higher numbers of capillaries (P = 0.02), whereas the lower drug dose gave no significant increase (Fig 6). Stents loaded with 300 μg 6-MP induced 30% more capillaries than polymer-only stents (P = 0.02).

## Discussion

Restenosis has been the primary obstacle after balloon angioplasty and stent implantation. Drug-eluting stents (DES) have dramatically reduced restenosis, but introduced the risk of stent thrombosis, sentencing patients to long-term anti-platelet medication. Restenosis is mainly caused by elevated SMC proliferation and exacerbated by infiltration of inflammatory cells. However, stent thrombosis is potentiated by stent polymer or metal exposed to the blood, highlighting the importance of endothelial cell growth and survival after stent placement. To address these cell-specific challenges, we devised stents coated with a 6-MP eluting bioresorbable polymer coating. These novel 6-MP eluting stents were tested in the rabbit iliac artery and were shown to consistently inhibit restenosis and decrease inflammation after 4 weeks, and to promote endothelial cell coverage of the stent after 1 week.

The inhibition of restenosis in rabbit iliac arteries found in the present study is consistent with previous findings on the protective function of 6-MP in vascular disease. Most relevantly, local 6-MP delivery attenuated neointima and SMC-rich lesion formation through activation of the nuclear receptor Nur77 in mice [17]. In the murine carotid artery ligation model Nur77 was shown to inhibit neointima formation involving increased expression of p27^Kip1^ [15]. Accordingly, Nur77 was shown to play a central role in inhibition of neointima hyperplasia after balloon injury in rat carotid arteries [30,31]. In one of these rat studies, the inhibition was established by induction of SMC apoptosis [30], however this was not observed in the present study or in previous mouse studies [14]. Cleaved-caspase-3 expression was low and not different between the groups. More likely, the difference in restenosis may be explained by induction of the cyclin-dependent kinase inhibitor p27^Kip1^. Stents loaded with 300 μg 6-MP showed increased p27^Kip1^ expression in intimal SMC, in accordance with previous studies showing the relation between p27^Kip1^ and neointima hyperplasia [32,33], and between p27^Kip1^ and 6-MP or Nur77 [15–17].

In monocytes and macrophages, Nur77 expression is elevated in response to atherosclerotic factors [34]. Nur77 was shown to reduce inflammatory responses and lipid loading [20,22,25,34,35]. In arterial remodeling, Nur77 attenuated macrophage accumulation [36]. 6-MP reduces the inflammatory response of macrophages albeit in a Nur77-independent way [37,38]. In agreement, our results show a reduction of macrophage infiltration in arteries with 6-MP eluting stents compared with the polymer only stents. A direct correlation between restenosis rate and macrophage infiltration was not observed (data not shown), suggesting that the lower inflammation was not the main cause for the observed reduction in restenosis.

The reduction of restenosis obtained with the 6-MP eluting stents in the present study may not be as high as both rapamycin- and paclitaxel-eluting stents, demonstrating a stronger reduction in restenosis in rabbit iliac arteries [39,40]. However, the main complication of those and other currently used DES has been the delayed healing, putting patients at risk for stent thrombosis [6,40]. This complication is clearly absent in our 6-MP-eluting stents. Even as early as one week after implantation, struts are covered by a monolayer, as opposed to the rapamycin-eluting stent. All metal struts of this Cypher stent were completely exposed, one week after implantation. The fast coverage of stent struts diminishes the risk of thrombosis, making the stent tested in the present study a promising therapeutic option. The beneficial coverage in the present study is in accordance with previous studies on the effect of 6-MP on endothelial cells. Administration of 6-MP stimulated endothelial cell survival, increased capillary sprouting and reduced inflammatory response in endothelial cells [18,38]. In the present study this mechanism is further confirmed by the increased number of capillaries with the high 6-MP dose. The increase in capillary number suggests good penetration of the drug in the vessel wall, and stresses its cell-specific functioning.

This study is the first to show the potential of Nur77 as a therapeutic target in the prevention of in-stent restenosis. However, the maximal duration of stent implantation was 4 weeks. Although this is a validated time frame in rabbits, it limits the conclusions on long-term safety. This period is for example insufficient to study neo-atherosclerosis, which emerges as an important factor in long-term vascular response to stent implantation. Another limitation of this proof of concept study is that strut coverage was evaluated qualitatively in only a limited number of samples. Overall, the present results suggest that a higher 6-MP dose may improve performance beyond what is seen in this study, so a broader exploration of the effective dose range may be warranted. Furthermore, the results should be replicated in another animal model, preferably in porcine coronary arteries, to study safety and efficacy on the longer term and to bring this therapy closer to the clinic.

## Conclusions

In summary, we demonstrate that novel stents coated with a bioresorbable polymer coating eluting 6-MP inhibit restenosis and attenuate inflammation, while stimulating endothelial coverage. The 6-MP-eluting stents demonstrate that inhibition of restenosis without leaving uncovered metal is feasible, bringing stents without risk of late thrombosis one step closer to the patient.

## Supporting Information

S1 ARRIVE Checklist (DOCX)
