# Supplementary material for: Stents Eluting 6-Mercaptopurine Reduce Neointima Formation and Inflammation while Enhancing Strut Coverage in Rabbits
Source: PLoS One. 2015 Sep 21;10(9):e0138459. doi: 10.1371/journal.pone.0138459 (PMC4577071; doi:10.1371/journal.pone.0138459)

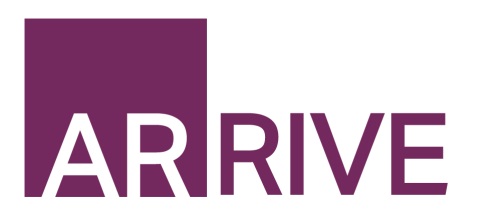


The ARRIVE Guidelines Checklist

Animal Research: Reporting In Vivo Experiments

Carol Kilkenny^1^, William J Browne^2^, Innes C Cuthill^3^, Michael Emerson^4^ and Douglas G Altman^5^

*^1^The National Centre for the Replacement, Refinement and Reduction of Animals in Research, London, UK, ^2^School of Veterinary Science, University of Bristol, Bristol, UK, ^3^School of Biological Sciences, University of Bristol, Bristol, UK, ^4^National Heart and Lung Institute, Imperial College London, UK, ^5^Centre for Statistics in Medicine, University of Oxford, Oxford, UK.*

|  | | ITEM | RECOMMENDATION | Section/ Paragraph |
| --- | --- | --- | --- | --- |
| 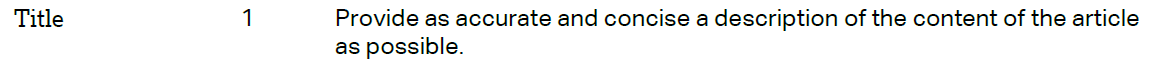 | | | Title |  |
| 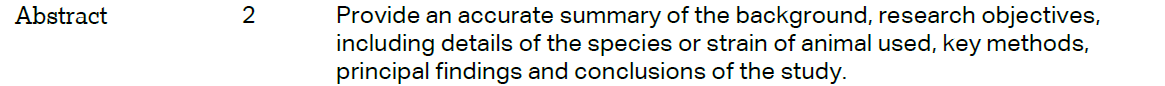 | | | Abstract |  |
| INTRODUCTION | | |  |  |
| 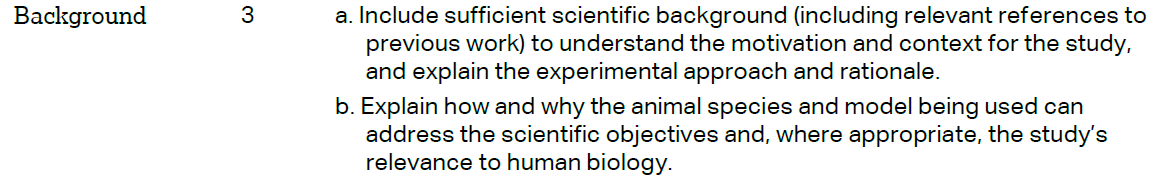 | | | Par 1-3  Par 2-3 |  |
| 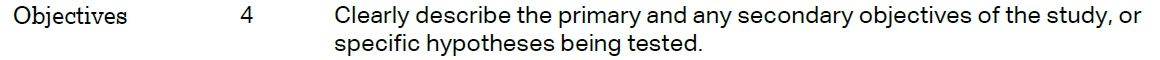 | | | Par 3 |  |
| METHODS | | |  |  |
| 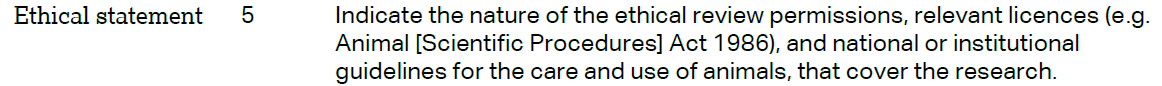 | | | Methods: Animal model |  |
| 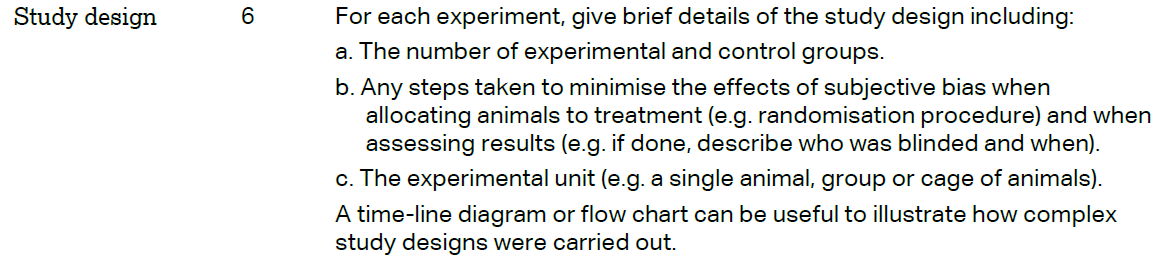 | | | Methods: Animal model; Surgical procedure |  |
| 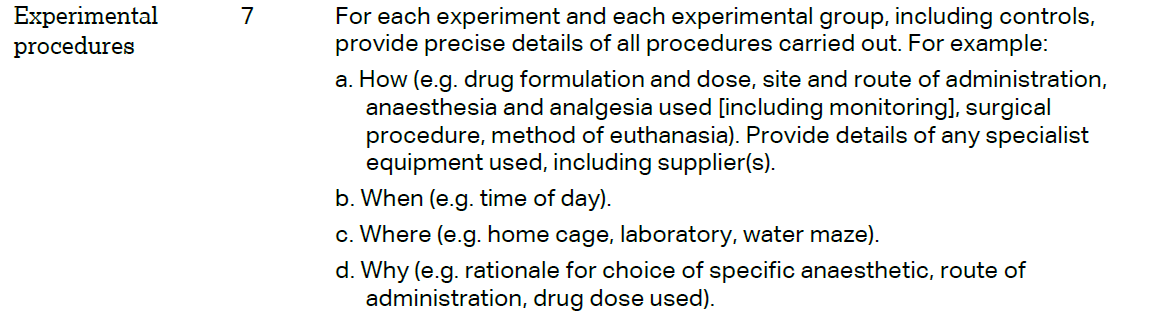 | | | Methods: Animal model; Surgical procedure |  |
| 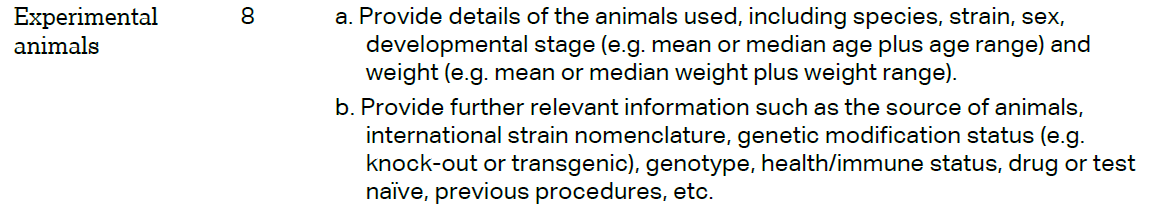 | | | Methods: Animal model; Surgical procedure |  |

The ARRIVE guidelines. Originally published in *PLoS Biology*, June 2010^1^

| 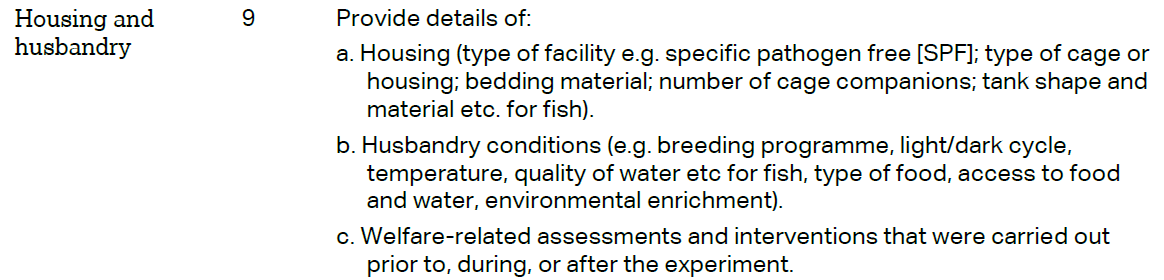 | Methods: Animal model; Surgical procedure | |
| --- | --- | --- |
| 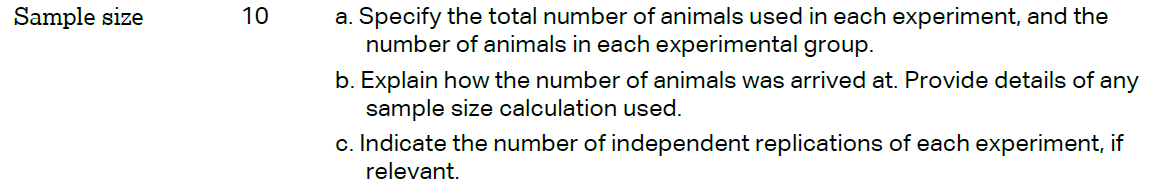 | Methods: Animal model; Surgical procedure | |
| 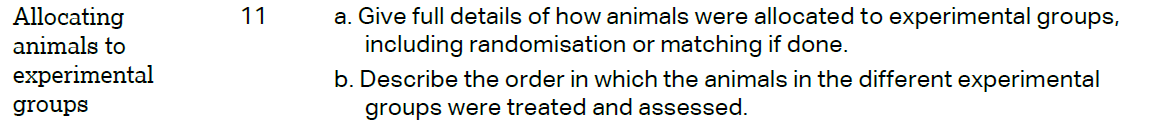 | Methods: Animal model | |
| 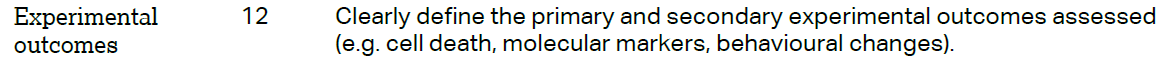 | Methods: Animal model; Surgical procedure | |
| 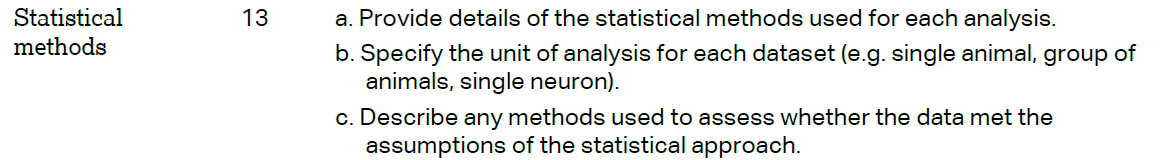 | Methods: Statistical analysis | |
| RESULTS |  | |
| 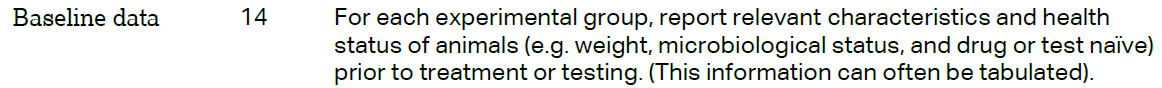 | Results: Paragraph 2 | |
| 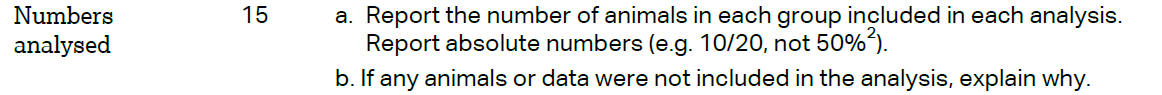 | Results: Paragraph 2 | |
| 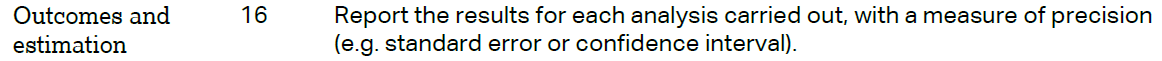 | Results: Paragraph 2-6 | |
| 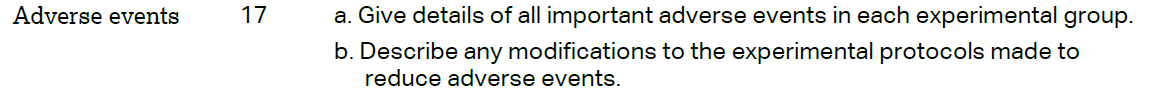 | Results: Paragraph 2 | |
| DISCUSSION |  | |
| 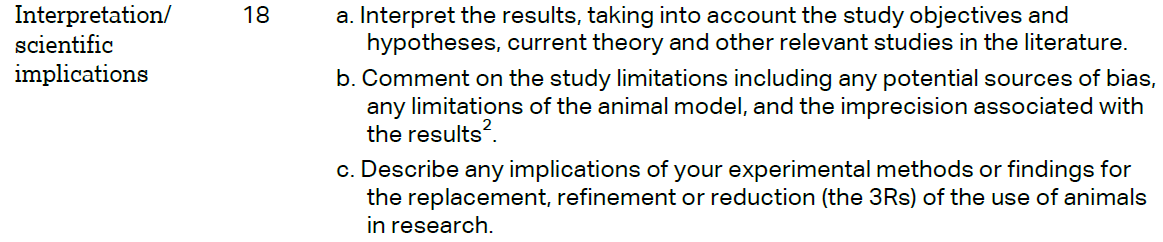 | Discussion, Paragraph 1-2 | |
| 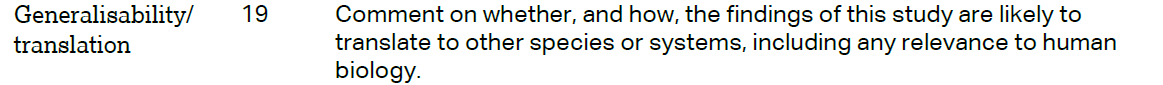 | Discussion, Paragraph 2-3 | |
| 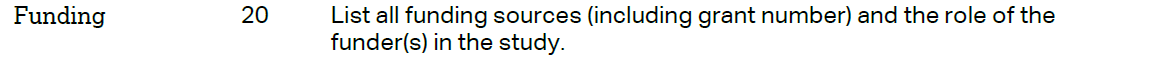 | | Funding |


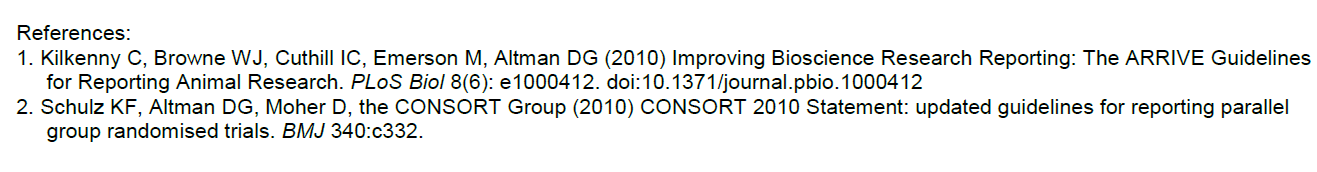

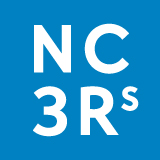

Supplement: S1 ARRIVE Checklist — (DOCX) [file pone.0138459.s001.docx]
